# Bacterial Communities: Interactions to Scale

**DOI:** 10.3389/fmicb.2016.01234

**Published:** 2016-08-08

**Authors:** Reed M. Stubbendieck, Carol Vargas-Bautista, Paul D. Straight

**Affiliations:** ^1^Interdisciplinary Program in Genetics, Texas A&M University, College StationTX, USA; ^2^Department of Biochemistry and Biophysics, Texas A&M University, College StationTX, USA; ^3^Department of Plant Pathology and Microbiology, Texas A&M Agrilife Research, WeslacoTX, USA

**Keywords:** bacterial communities, biodiversity, competition, ecology, interactions, microbiota, scaling, syntrophy

## Abstract

In the environment, bacteria live in complex multispecies communities. These communities span in scale from small, multicellular aggregates to billions or trillions of cells within the gastrointestinal tract of animals. The dynamics of bacterial communities are determined by pairwise interactions that occur between different species in the community. Though interactions occur between a few cells at a time, the outcomes of these interchanges have ramifications that ripple through many orders of magnitude, and ultimately affect the macroscopic world including the health of host organisms. In this review we cover how bacterial competition influences the structures of bacterial communities. We also emphasize methods and insights garnered from culture-dependent pairwise interaction studies, metagenomic analyses, and modeling experiments. Finally, we argue that the integration of multiple approaches will be instrumental to future understanding of the underlying dynamics of bacterial communities.

## Introduction

Bacterial communities vary in their species composition, niches occupied, and influence on different environments. Based on these complexities, communities defy a single fundamental definition. Rather, they represent fascinating examples of interactive processes that differ with ecological scale. The complications in defining and characterizing communities are reflected in the early history of microbiology. In the late 1800s Robert Koch revolutionized the field of microbiology by pioneering his methods to establish causality between a microorganism and disease (Koch, 1876). Even to this day Koch’s postulates remain the “gold standard” to associate microbes to disease or any other phenomenon of interest. Inspired by Koch’s reductionist approach, the vast majority of research over the past one hundred years has investigated the growth and physiology of microbes grown in pure culture. Studying single species of bacteria axenically was essential for birth of modern biochemistry and molecular biology and remains important to this day. However, even as early as the 1870s microbiologists including Louis Pasteur reported phenomena resulting from interactions of bacteria existing in multispecies communities (Pasteur, 1877). Bacteria are social organisms that interact extensively within and between species all while responding to external stimuli from their environments. Indeed, the ability to perceive neighboring cells and the environment is often reflected in the content of bacterial genomes. Recently, the construction of *Mycoplasma mycoides* JCVI-syn3.0, a bacterium with a minimal genome containing only 531 kilobase pairs and 473 genes was reported (Hutchison et al., 2016). When compared to the genome of a natural, soil bacterium *Myxococcus xanthus*, which contains 9.14 megabase pairs and 7388 protein coding genes (Goldman et al., 2006) the genome of *M. mycoides* JCVI-syn3.0 is miniscule. While JCVI-syn3.0 inhabits rich, complete media in the laboratory, *M. xanthus* competes in its environment and requires a large number of genes for signaling systems to interpret changing environmental conditions and the presence of competitors. In fact, many organisms, including two of our best studied model species *Bacillus subtilis* and *Escherichia coli*, contain large numbers of genes deemed “non-essential”. However, many of these genes may be absolutely critical to survival when bacteria are faced with competitors. As an old adage states: “no microbe is an island”. Thus, to truly understand a bacterial species it must be placed within its ecological context including the other members of its community. Communities are the collection of organisms that interact with each other and occupy the same physical location. For macroscopic organisms it is easy to draw physical boundaries and envision a community as the assemblage of fish in a pond or insects living in a tree. In contrast to schools of fish or hives of bees, bacterial communities are often more difficult to delineate because of their size relative to the environment.

**FIGURE 1:**
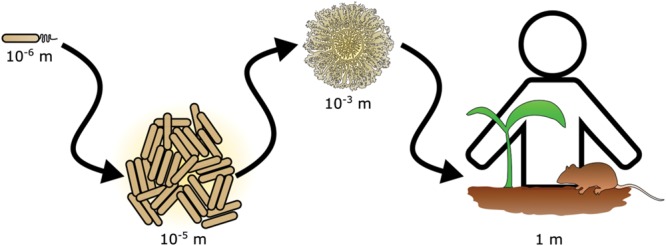
**Scaling in bacterial communities.** Bacterial communities range in scale from single cells to multicellular aggregates and colonies. The action of bacterial communities extends further affecting tissues and ultimately entire hosts.

The importance of scale is a recognized issue in ecology (Levin, 1992). In particular, scaling has important ramifications in the study of bacterial ecology (Green and Bohannan, 2006). Interactions that occur between bacteria in the range of single cells or small communities often have consequences for life at scales that span many orders of magnitude (**Figure 1**). For example, the action of photosynthetic cyanobacteria led to the oxygenation of our atmosphere and enabled the evolution of macroscopic organisms, including animals (Ohno, 1997). Bacterial communities living in and on plants and animals have powerful influences on health of the host. Historically, the experimentally accessible bacterial community was the colony or culture in laboratory media, but new technological advances have enabled the investigation of bacterial communities and their effects at multiple scales. For instance, within an animal’s gastrointestinal tract, bacteria may exert effects on other species that are separated by meters of intestine (Barman et al., 2008). However, at the opposite extreme non-motile bacteria on opposite sides of the same soil particle may never encounter each other (Carson et al., 2010). These examples highlight an obstacle in studying many bacterial communities *in situ*: due to scaling effects, defining the physical boundaries and properly sampling a bacterial community is challenging.

To understand a bacterial species, it must be placed into an ecological context, but doing so is challenging. Consequently, we use both biological and computational models to study bacterial communities. In this review we focus on current trends in understanding bacterial communities with an emphasis on bacterial competition and highlight innovative approaches that are used to experimentally characterize communities. We will begin by discussing different factors that influence how a bacterial community is structured. Then we will highlight several culture-dependent studies and technologies used to investigate bacterial competition. Next we will discuss culture-independent methods to study bacterial communities within the context of hosts. Lastly, we will discuss the intersection of competition and biodiversity as predicted by computational models. We will end with a brief discussion as to how these different approaches can complement each other and provide a more thorough understanding of bacterial communities.

## Bacterial Interactions Influence Community Structure

### Bacteria Preferentially Colonize Microenvironments that are Compatible with their Metabolic Strategies

Within a bacterial community cells of different species are not homogenously distributed, they are instead patterned by their interactions with neighboring cells and the abiotic environment according to their metabolic and physiological needs. Bacterial growth rate is shaped by natural selection in response to resource conditions over their ancestor’s history (Maitra and Dill, 2015). Since no cell encounters all types of situations and because carrying unused genes burdens a cell with extra metabolic costs, bacterial genomes become adapted for growth within specified environments according to natural selection. Further, because bacteria do not typically occur in axenic culture, their metabolic needs have been influenced by the metabolic functions of their neighboring cells. Therefore in addition to shifting environmental conditions, bacteria have also adapted to growth with neighbors (Freilich et al., 2009, 2011). In a simple case, two species located within the same community compete with each other for available resources. This form of competition is known as exploitation, wherein one species prevents its neighbor access to resources either by consuming or sequestering them (Birch, 1957).

When bacteria are grown in multispecies communities they employ “high risk, high reward” strategies, which are reflected in their specific metabolic adaptations. This concept arises from models of bacterial growth. Bacteria undergoing constant, (i.e., nutrient-insensitive) growth will always outcompete neighbors whose growth rate is dependent upon the external environment (i.e., nutrient-sensitive) (Mao et al., 2015). However, we do not typically observe nutrient-insensitive growth patterns. Indeed, no bacterial species has a constant growth rate under all types of changing conditions. Compared to constant growth, cells that better exploit their environment will potentially produce more progeny. Additionally, resources in the environment may fluctuate on short timescales. However, by definition nutrient-sensitive growth strategies are susceptible to the environment, and cells encountering suboptimal environments, relative to their metabolic adaptations, will be outcompeted. In summary, growth and occupancy of any community is defined by flexible metabolic strategies according to the external environment.

Heterogeneous distributions of metabolism and nutrients set up microenvironments, which become enhanced by diffusion occurring over multiple directions with respect to different resources. The drivers of these microenvironments are resource gradients including nutrients, pH, physical space, reducing agents, and terminal electron acceptors (e.g., Rousk et al., 2010; Goldfarb et al., 2011; De Weirdt and Van de Wiele, 2015). The effects of microenvironments on communities are detectable within laboratory models such as biofilms of bacterial cells (Stewart and Franklin, 2008). However, bacteria live in complex natural environments. For instance, the human GI tract has a total length on the scale of meters and is subdivided into regions like the stomach or the intestines, which are even further divided into the small intestine and colon. Each region is composed of different tissue types, which influence the external environments that bacteria experience. Furthermore, as meals transit through the GI tract, bacteria in different regions are exposed to different amounts and types of nutrients (Donaldson et al., 2016). All of these factors contribute to the complexities of community structure at differing spatial and temporal scales, as each bacterial species adopts a different growth strategy with different nutritional requirements.

**FIGURE 2:**
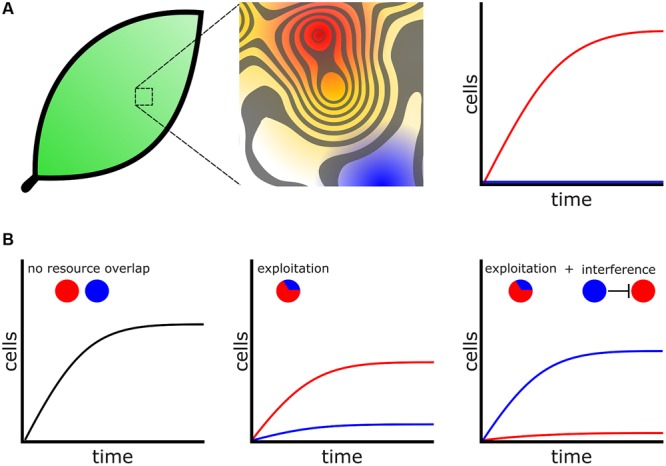
**Bacteria compete for favorable microenvironments. (A)** (Left) Environments, including biological tissues such as leaf surfaces, are heterogeneous with respect to favorable microenvironments. (Middle) Magnified leaf surface shown as a contoured fitness landscape. Higher peaks represent more favorable microenvironments with increased carrying capacity. For assistance in interpretation the contour surface has been overlaid onto a heat map. Red represents the most favorable microenvironments while blue represents poor microenvironments. (Right) Cells that colonize favorable microenvironments (Red) produce many progeny whereas cells occupying poor microenvironments fail to proliferate (blue). **(B)** Bacteria engage additional mechanisms to colonize microenvironments. (Left) Two species, represented by colored circles, which do not overlap with respect to physical location or resource usage will not compete. (Middle) When cells are in conflict for resources the species that is better adapted to exploit those resources will proliferate while the poorer exploiter struggles. (Right) A poor exploiter and use additional competitive mechanisms, such as interference, to prevent a better exploiter access to a favorable microenvironment.

In addition, community structure affects the fitness of bacterial cells and these influences are present in every environment that bacteria inhabit. For instance, leaf surfaces are hostile environments comprised of many heterogeneous microenvironments with respect to available resources (**Figure 2A**; Lindow and Brandl, 2003). Measurements from a clonal population of *Erwinia herbicola* cells inoculated evenly over the total leaf surface revealed that most cells fail to divide, but a small population of cells managed to divide five times or more in the same period of time. This dependency of growth on locale illustrates the heterogeneous carrying capacities that exist among microenvironments on the surface of a single leaf (Remus-Emsermann et al., 2012) and is probably the case for many natural environments. The difference between cells colonizing an optimal versus a poor microenvironment may be the difference between proliferation and extinction. Currently, the features that determine the size and structure of an optimal or poor microenvironment are not well understood. However, some technological innovations now provide a window into microenvironment structure. Recently the use arrays of microfabricated wells revealed that bacterial growth is stochastic when cells are constrained to growth in small microwells with diameters less than 100 μm, because of the variability in the inoculation of these wells. This observation suggests that a constrained space can greatly influence the growth and assemblies of communities (Hansen et al., 2016). Microwells may become an important model for studying how heterogeneous microenvironments influence bacterial growth and competition.

For a given “high risk, high reward” metabolic strategy to remain viable, bacteria must engage additional competitive mechanisms to acquire and defend territory (Liu et al., 2011; **Figure 2B**). One concept arising from modeling is that bacteria species with similar metabolic adaptations will compete for the same heterogeneously distributed favorable microenvironments (Freilich et al., 2011). Bacteria may inhibit or kill competitors and prevent invasion into their territories through interference competition, releasing diffusible antagonists such as toxins or antibiotics that create inhospitable zones for competitors (Stubbendieck and Straight, 2016). Individuals of a species may also collectively operate and use developmental functions to exclude their competitors, which enables that species to better exploit resources available in a microenvironment (Hibbing et al., 2010). These types of interactions drive collective functions seen in competitive environments. For example, in a bacterial colony oxygen levels decrease with increasing depth (Hibiya et al., 2004). Obligate aerobes will preferentially distribute themselves toward the more oxygen-rich colony surface. As there is only limited surface area in direct contact with oxygen there are an inadequate number of microenvironments with optimal oxygen concentrations. Consequently, under laboratory conditions *Pseudomonas fluorescens* cells rapidly acquire mutations that allow them to better engage in exploitation competition for the oxygen rich surface. By producing more extracellular polysaccharides (EPS), exploiters can “push” themselves to the surface and outcompete their isogenic siblings (Kim et al., 2014). In another example, surface localization is so important for aerobic bacteria that in *B. subtilis* biofilms EPS producers undergo controlled cell death, which relieves lateral pressure in the colony causing it to buckle and push nearby cells toward the colony surface (Asally et al., 2012).

One of the more clearly delineated examples of resource driven community level interactions is seen in the oral community. Under anaerobic conditions two facultative anaerobes, a commensal species *Streptococcus sanguinis* and a cariogenic species *Streptococcus mutans*, coexist in dental plaque (Kreth et al., 2005, 2008). Under aerobic conditions *S. sanguinis* oxidizes pyruvate and produces hydrogen peroxide (H_2_O_2_) as a byproduct, which inhibits the growth of competitor *S. mutans* (Kreth et al., 2008). Mutants of *S. mutans* that are unable to produce glutathione, which can detoxify H_2_O_2_, are more sensitive to competition with *S. sanguinis* (Zheng et al., 2013). Intriguingly, *S. mutans* glutathione mutants also increase EPS production (Zheng et al., 2013), which has been shown to protect *E. coli* cells from H_2_O_2_ (Chen et al., 2004). These examples, including *P. fluorescens* and *B. subtilis*, demonstrate the connection between extracellular resources and community-level changes in cellular functions (e.g., EPS production) to colonize optimal microenvironments.

### Competitors in a Community Influence Metabolism

Bacteria living within communities often have access to a wider range of nutrients than single cells living in isolation from other species (Ponomarova and Patil, 2015). This community service is provided by cells that facilitate the growth of others or through obligately mutualistic metabolism (also called “metabolic syntrophism”), resulting in complex and multispecies biochemical dependencies (Prokopenko et al., 2013). Metabolic syntrophism suggests that cells cooperate because different species possess complementary biochemical pathways needed to liberate nutrients from the environment (Morris et al., 2013). For instance, the oxidative degradation of alkanes to acetate requires the concerted effort of two species, *Smithella* and *Marinobacter* (Gray et al., 2011). Also, methanotrophic archaea and sulfate-reducing bacteria aggregate together and degrade methane within marine methane seeps through associations mediated by nitrate (Pernthaler et al., 2008; Green-Saxena et al., 2014). With these and other examples it is clear that syntrophic interactions are important for communities, however, the origins of these interactions may have arisen in competitive contexts.

Competition for adaptive gene loss may form the foundation for syntrophic interactions resulting in multispecies biochemical dependencies. Natural selection favors cells that are best adapted for growth in the conditions they encounter (see above). During DNA replication the entire genome must be duplicated with each successive base imparting more metabolic cost to the cell. Selection therefore favors bacteria that undergo genome streamlining while maintaining necessary metabolic functions that are not provided by the community at large (Koskiniemi et al., 2012; Lee and Marx, 2012; Morris et al., 2012). This is because cells that adaptively lose genes are able to invest more energy into other aspects of metabolism including growth and division. This model, also known the “Black Queen Hypothesis” was recently described with an example of *Prochlorococcus* in communities with *Synechococcus* (Morris et al., 2012). In mixed communities, *Synechococcus* detoxifies H_2_O_2_ via the action of catalase-peroxidase (KatG). The adaptive loss of the *katG* gene from *Prochlorococcus* allows it to engage in a unique form of exploitation competition with *Synechococcus*. If the population of *Synechococcus* also loses the *katG* gene then the entire community becomes sensitive to H_2_O_2_, which is rapidly produced during photooxidation of carbon, resulting in the death of both species. The *katG* gene is maintained in the population because there is selection against *Synechococcus* undergoing adaptive loss of the same function and leading to mutually assured destruction of the community. This example and others suggest that cooperative community interactions may be born out of selection under competitive pressures (Morris, 2015). Subsequently, once an essential function is no longer redundant within a community, selection may favor specialization of cells to reduce biochemical conflicts and improve their fitness (Johnson et al., 2012). As a consequence of functional specialization, cells adopt new growth strategies, which affect how they interact with available microenvironments and their neighbors.

### Competition for Physical Space Influences Community Structure

In the macroscopic world, leafy plants can produce large leaves and prevent shorter plants access to sunlight and outcompete them (Walker et al., 1988). Analogously, bacteria can also use growth to gain access to space and starve their competitors by colonizing large areas, as opposed to positioning themselves within a favorable microenvironment. By colonizing larger surface areas, a bacterial species increases the probability that it will occupy a larger fraction of favorable microenvironments, even if the majority of cells ultimately inhabit suboptimal microenvironments. Consequently, occupying more total surface area is advantageous by preventing competitors from accessing the same microenvironments.

Motile and non-motile bacteria employ different strategies to compete for space. In the former case, motile cells guided by chemotaxis rapidly advance over a permissive surface and guide themselves to favorable microenvironments. Motile populations may deposit cells as they trespass different microenvironments. Whether individual cells proliferate or stagnate depends on the favorability of the microenvironment. Though many available microenvironments may be suboptimal, a population in motion has a higher probability of occupying the most favorable niches. As populations gain a foothold on new territory, kin selection mechanisms may help to ensure the success of siblings and exclusion of unrelated competitors (Vos and Velicer, 2009; Alteri et al., 2013; Stefanic et al., 2015). In contrast, non-motile bacteria must grow to compete for physical space. As an example, *E. coli* was seeded onto high percentage agarose pads at very low cell density to promote microcolony formation from single cells (Lloyd and Allen, 2015). In this format, the shape and size of developing colonies was dictated by the distribution of neighboring microcolonies. Using a mathematical model to eliminate growth lag as a variable, Lloyd and Allen showed that mechanical interactions generate “pushing” forces between expanding microcolonies. A pushing force influences the final colony shape, and thus the ability of non-motile bacteria to access and inhabit favorable microenvironments. Thus, for both motile and non-motile bacteria the competition for physical space is affected by the availability of favorable microenvironments and the distribution of cells in a community.

Unlike the above example focused on colonization from single cells, bacteria in natural environments often exist as multicellular aggregates that are sometimes attached to relatively large particles (Alldredge and Silver, 1988). Multicellular aggregates have the potential to inoculate a high number of bacteria in a new environment. In nature this occurs, for example, when large pieces are broken off of a biofilm (Stoodley et al., 2001a,b). Inoculum size and growth history of a community often determines the competitive success of community members. For instance, the number of competitors on a colonized surface impacts the fitness of *P. aeruginosa* cell aggregates under oxygen limitation. At low competitor density, the aggregate cells are less fit than their surface-associated single cell competitors. At higher competitor density on the other hand, the aggregate cells are more fit than their competitors (Kragh et al., 2016). The relation between density and fitness is thought to arise because single cell competitors have a higher surface area to volume ratio (SA/V) than cells contained in aggregates. Under these conditions the average single cell has better access to resources than the average aggregate cell. Additionally, competition may occur between cells inside the aggregate and lower the overall fitness of the aggregate (Rendueles and Ghigo, 2015). However, at higher competitor density the single cells are packed more tightly together and their effective SA/V decreases, which reduces their fitness relative to the average aggregated cell.

In a complementary approach, mathematical modeling predicts that the shape of an aggregate affects its fitness depending upon competitor density. In the absence of competitors, aggregates with smaller incident angles, relative to their surface, are more successful because their SA/V is maximized. However, in the presence of surface-associated, single-cell competitors, aggregates with higher incident angles, and rounder shapes become more successful (Melaugh et al., 2016). In addition to the decreased benefits of growth as single cells due to dwindling access to resources, cells at the top of the aggregate are far removed from competition and the conditions of limiting oxygen at the developing biofilm surface (Kragh et al., 2016). As a consequence of this resource disparity, under highly competitive conditions in developing biofilms a disproportionate number of cells are descended from aggregate cells and not single cell competitors (Kragh et al., 2016).

In summary, bacteria in communities are influenced by external factors including available microenvironments and the presence of different competitors. The spatial positioning of cells within a community reflects competition for optimal microenvironments and is modulated by an individual’s metabolic needs and the spatial structure of the community itself.

## Culture-Dependent Models for Bacterial Competition

Though a bacterial community may be comprised of large number of cells, bacteria likely interact at the scale of single cells or multicellular aggregates. The dynamics of the community at large are thus determined by interactions that occur between pairs of individual cells residing within the community. Using models of bacterial competition is a proven approach to address fundamental questions regarding competitive mechanisms, which often take the form of macroscopic bacterial colonies competing on an agar plate. Though artificial, by observing macroscopic colonies we gain insight into competitive mechanisms that bacteria use at single cell levels. For instance, Alexander Fleming’s famous laboratory observation that *Penicillium chrysogenum* inhibited *Staphylococcus aureus* growth may be one of the first examples of interference competition investigated by this method (Fleming, 1929). The agar plate was subsequently adopted for screening antibiotic compounds and it remains an invaluable tool for investigating competition (Lewis, 2013). The outcomes of competition on an agar plate are often manifested in visible phenotypes including developmental defects, growth inhibition, lysis, motility, and pigment production (e.g., Kerr et al., 2002; Be’er et al., 2009; Teasdale et al., 2009; Garbeva et al., 2011; Shank et al., 2011; Cude et al., 2012; Traxler et al., 2012, 2013; Alteri et al., 2013; Koch et al., 2014; Wang et al., 2014; Hol et al., 2015; Powers et al., 2015).

**FIGURE 3:**
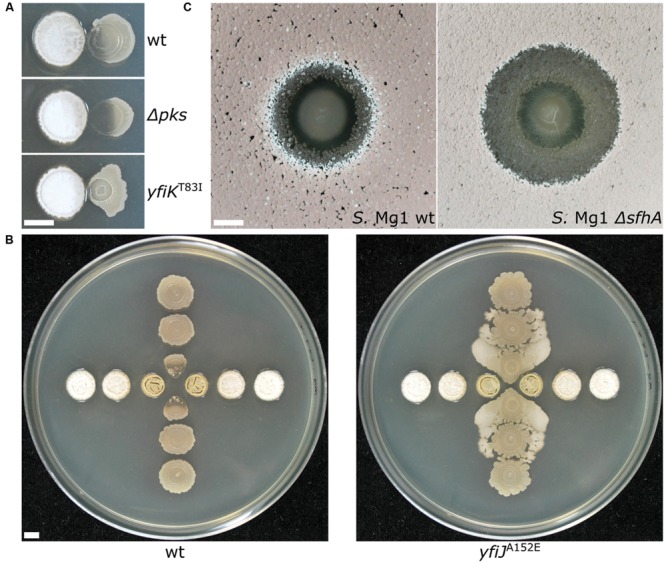
**Changing the dynamics of bacterial competition uncovers new competitive mechanisms. (A)** (Top) *Streptomyces* sp. strain Mg1 (*S.* Mg1) (left) releases linearmycins and lyses *Bacillus subtilis* (right) when both organisms are grown next to each other on an agar surface. (Middle) Strains of *B. subtilis* that are unable to produce the specialized metabolite bacillaene (*Δpks*) are hypersensitive to lysis by *S.* Mg1. (Bottom) A mutation *yfiK*^T83I^ that activates a two-component system causes *B. subtilis* to become linearmycin resistant. **(B)** *S.* Mg1 is plated in the horizontal direction while *B. subtilis* is plated in the vertical direction. (left) wild type *B. subtilis* is lysed by *S.* Mg1 as above but a linearmycin resistant (right) mutant of *B. subtilis* engages motility in response to the presence of *S.* Mg1. **(C)** *B. subtilis* plated as a spot onto a lawn of *S.* Mg1. The wild type *S.* Mg1 (left*)* undergoes sporulation as evident by the salmon coloration but the sporulation of a mutant unable to produce surfactin hydrolase (*ΔsfhA*) is inhibited. All images were taken after 72 hours of co-incubation. The scale bar is 5 mm. The panels in **(A)** were reproduced from Stubbendieck and Straight (2015) under the terms of the Creative Commons Attribution License.

Our own experience using *B. subtilis* and *Streptomyces* spp. in different formats reveals variable patterns and functions of bacterial competition. Importantly, changing the competitive dynamics between these organisms by using different species or mutants of *Bacillus* and *Streptomyces*, or changing plating formats has continued to uncover new mechanistic insights into functions of secreted enzymes and specialized metabolites (SMs) in bacterial competition. For example the *B. subtilis* produced SM bacillaene, originally identified as a translation inhibitor (Patel et al., 1995), is involved in a suite of functions with respect to different competitors. Bacillaene also inhibits the growth of *Streptomyces avermitilis* (Butcher et al., 2007), interferes with production of pigmented prodigiosin *Streptomyces coelicolor* (Straight et al., 2007) and *Streptomyces lividans* (Vargas-Bautista et al., 2014), and is involved in defense against consumption by *Myxococcus xanthus* (Müller et al., 2014) and linearmycin-induced lysis by *Streptomyces* sp. strain Mg1 (*S.* Mg1) (Barger et al., 2012; Stubbendieck and Straight, 2015; **Figure 3A**). In the latter case, linearmycin-resistant *B. subtilis* mutants also revealed competitive functions for a previously uncharacterized two-component signaling system that controls expression of the genes for an ATP-binding cassette transporter, which is involved in both specific linearmycin resistance and causes morphological changes in *B. subtilis* with regards to motility and colony development (Stubbendieck and Straight, 2015; **Figure 3B**).

**FIGURE 4:**
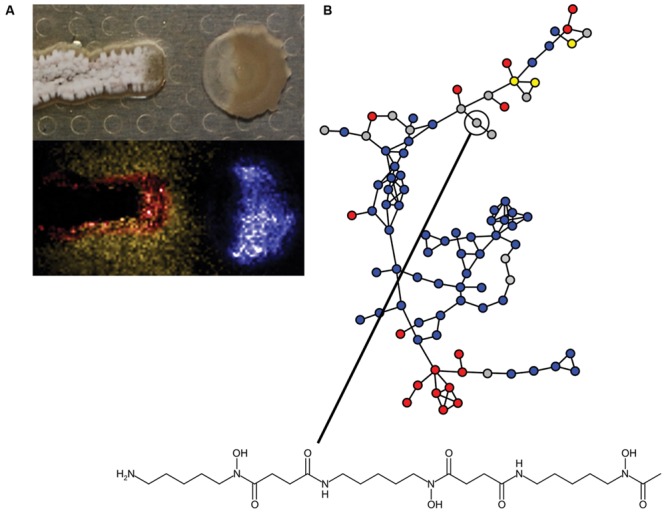
**Insights into bacterial competitive metabolism through mass spectrometry. (A)** (Top) *Streptomyces* sp. strain Mg1 (*S.* Mg1) (left) releases linearmycins and lyses *Bacillus subtilis* (right) as in **Figure 3A**. (Bottom) The distribution of metabolites produced by both organisms is mapped by imaging mass spectrometry (IMS). The false-colored extracted ion image shows the distribution of diffuse chalcomycin A produced by *S.* Mg1 (yellow), a *B. subtilis* colony marker polyglutamate (blue), and an unknown colony localized metabolite produced by *S.* Mg1 (red, *m/z* 972). **(B)** Mass spectral molecular networking data of competitions between *Streptomyces coelicolor* and other actinomycetes. Each node represents a metabolite identified in a mass spectrometer and the edges between nodes indicate chemical relationship as determined by aligned tandem MS/MS spectra. Blue nodes indicate metabolites produced by *S. coelicolor*, red nodes are metabolites produced by competitors, yellow nodes are metabolites produced by both *S. coelicolor* and competitors, and gray nodes are metabolites with variable behavior. A node corresponding to desferrioxamine B is indicated and its structure is shown. Mass spectral networking identified new variants of desferrioxamines that had not been previously reported. Panel **(A)** was adapted from Barger et al. (2012), Copyright © Springer Science+Business Media B.V. 2012, with permission of Springer. The data in panel **(B)** were reproduced from Traxler et al. (2013) under the terms of the Creative Commons Attribution License.

The information content of different pairwise interactions has been greatly enhanced by application of new technological innovations. For example, one such transformative technology is imaging mass spectrometry (IMS). Initially, IMS of bacterial competition was investigated using matrix assisted laser desorption ionization time-of-flight (MALDI-TOF) mass spectrometry (Yang et al., 2009). MALDI-TOF has previously been used clinically for the identification of bacteria based on their metabolite and proteome fingerprints (Singhal et al., 2015). However, for MALDI-TOF-IMS bacterial cultures are initially grown on an agar plate, which is subsequently covered in MALDI matrix and scanned over a two-dimensional area by programming a mass spectrometer to collect mass spectra at each X,Y with defined raster distance. The mass spectra are composited together into a spatially organized dataset such that the distribution of molecules produced by each organism can be mapped (Yang et al., 2009; Hoefler and Straight, 2014). MALDI-TOF-IMS has been used to investigate bacterial competition in a number of systems. Specific examples of the use of MALDI-TOF-IMS include revealing that *B. subtilis* produces more surfactin when it is cultured next to competing *S. aureus* (Gonzalez et al., 2011). Surfactin also inhibits sporulation of many *Streptomyces* species by antagonizing a morphogenetic peptide SapB, which is visually striking on an agar plate (Straight et al., 2006; Gaskell et al., 2012). MALDI-TOF-IMS of competitions between *B. subtilis* and *S.* Mg1 revealed that the latter produces a secreted hydrolase (SfhA) that specifically degrades surfactin and plipastatin produced by the former (Hoefler et al., 2012; **Figure 3C**). Additionally, MALDI-TOF-IMS of competitions between these same two organisms led to the identification of chalcomycin A produced by *S.* Mg1 and revealed patterns of many unknown SMs that may also be involved in competition (Barger et al., 2012; **Figure 4A**). As a final example, MALDI-TOF-IMS was used to identify thiocillins produced by *Bacillus cereus*, which induce biofilm formation of *B. subtilis* (Bleich et al., 2015).

More recently, IMS approaches using nanospray desorption electrospray ionization (nanoDESI) have been instrumental in circumventing some limitations of MALDI. Two major advantages of nanoDESI-IMS are 1) matrix is not necessary, which allows imaging of living cultures and 2) higher resolution and tandem mass analysis can be used to glean structural information from analyzed metabolites (Rath et al., 2013; Watrous et al., 2013). The latter feature has been instrumental in the development of mass spectral networking, which is used to visualize the chemical relationships among diverse metabolites produced by bacteria (Guthals et al., 2012). For example, nanoDESI and spectral networking revealed that the secreted metabolome of *S. coelicolor* responds idiosyncratically with respect to challenge by different competitors. Among the different SMs identified were new members of the desferrioxamine siderophore family (Traxler et al., 2013; **Figure 4B**). IMS and mass spectral networking have transformed our view of SM usage in bacterial competition.

While IMS experiments and morphological observations are useful approaches for understanding mechanisms in bacterial competition, other technologies have also enabled the investigation of bacterial communities on even smaller scales. At spatial scales relevant to natural bacterial competition, interactions likely occur between populations of cells in the range of 10^3^ cells or fewer. Bacteria can be studied at low numbers using conventional microscopy and agarose pads. For individual cells, exponential growth leads to high cell density in a few generations, obscuring microscopic observation. Also, nutrient and oxygen may become limiting and agarose pads dry out during extended incubation (Moffitt et al., 2012). Fortunately, micromanipulation and microfabrication techniques surmount some of the conventional limitations, enabling microscopy of bacterial interactions at more realistic population densities and at longer time scales. One approach to investigating bacterial interactions has been through the use of microfluidic devices that constrain the number of cells and the physical space in which they interact (reviewed in Rusconi et al., 2014). An example of such a device is a three-species community consisting of *Azotobacter vinelandii, Bacillus licheniformis*, and *Paenibacillus curdlanolyticus*, which is unstable under mixed culture conditions, leading to collapse with one species predominating. However, the three-species community is stable when each member is cultured in separate microfluidic chambers with limited nutrients to force syntrophy (Kim et al., 2008). Likewise, spatial separation via microfluidic chambers can stabilize synthetic bacterial communities with new function in bioremediation. Microfluidics enabled the formation of two-species community consisting of *Ralstonia metallidurans* and *Sphingobium chlorophenolicum*. If both species are separated then they can simultaneously detoxify mercury(ii) and pentachlorophenol, which are environmental contaminants that may colocalize. However, without spatial structure *S. chlorophenolicum* is unable to degrade pentachlorophenol in the presence of mercury due to its sensitivity (Kim et al., 2011).

Other examples illustrate the benefit of using microfluidic devices to study competitive bacterial interactions. For instance microfluidic devices have been used to investigate predation of *E. coli* by *Bdellovibrio bacteriovorus*. By using concentrator arrays Park et al. (2011) were able to observe predation of single cells of *E. coli* by single cells of *B. bacteriovorus* at a variety of predator-prey ratios and directly measure predation rates. Further, microfluidic devices containing arrays of structured microhabitats revealed the influence of environmental patchiness on prey *E. coli* escape and survival from *B. bacteriovorus* predation. Unlike batch culture where most *E. coli* is consumed by *B. bacteriovorus*, in structured microhabitats *E. coli* persists through geometric-assisted formation of biofilms (Hol et al., 2016). Structured microhabitats have also been used to study reproducible spatiotemporal patterns of *E. coli* populations competing for physical space. When populations of isogenic *E. coli* inoculated from opposite ends of a microfluidic device meet, they exchange diffusible signals and reflect each other, perhaps to avoid unnecessary attempts to colonize already occupied microenvironments (van Vliet et al., 2014). Finally, microfluidic devices have also been used to demonstrate that natural isolates of *P. aeruginosa* induce biofilm formation as a response to general damage inflicted by competitors (Oliveira et al., 2015).

**FIGURE 5:**
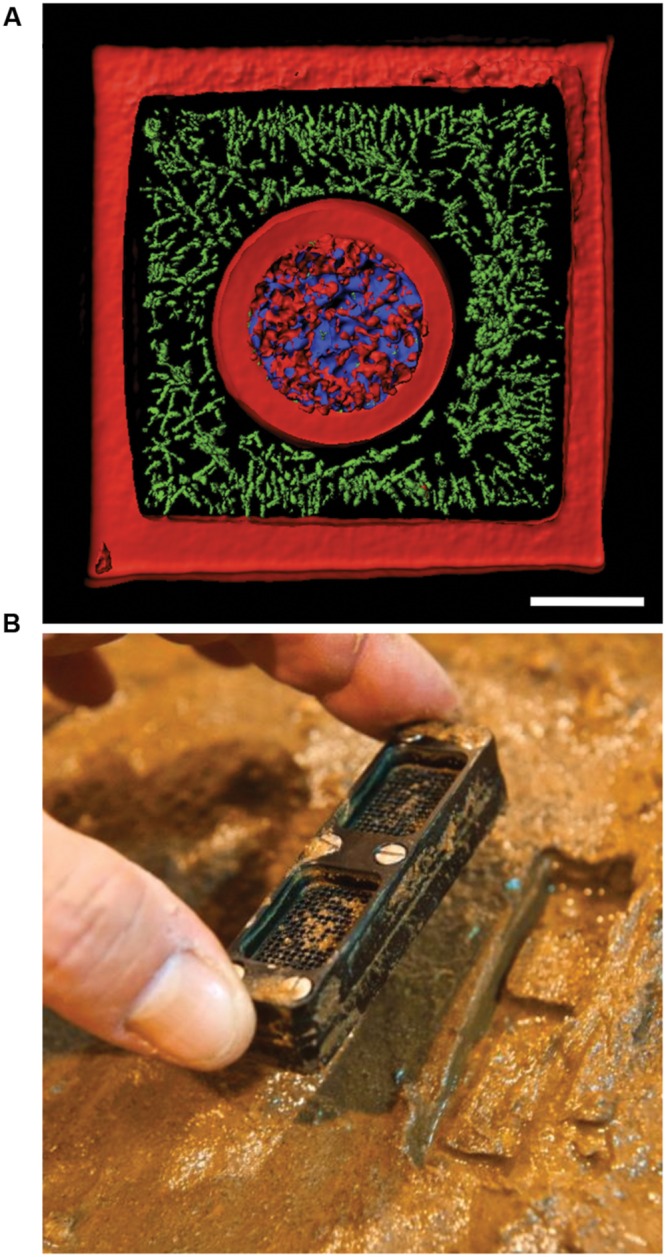
**New technologies to study bacterial communities. (A)** 3D printed bacterial community consisting of a shell of *Staphylococcus aureus* (blue) encased by *Pseudomonas aeruginosa* (green) encased in a crosslinked gelatin matrix (red). The scale bar is 10 μm. **(B)** Photograph showing an iChip after incubation in an environmental setting. Panel **(A)** was provided by Jodi Connell and Marvin Whiteley. Panel **(B)** was reprinted by permission from Macmillan Publishers Ltd (Ledford, 2015), Copyright © 2015, Rights Managed by Nature Publishing Group.

Concurrently with advances in microfluidic technologies has been the development of bacterial microcontainers. Dynamic mask-based multiphoton lithography (MPL) methods have been used to fashion bacterial microcontainers from bovine serum albumin (BSA) (Connell et al., 2010). Microcontainers are formed by using a laser to covalently crosslink BSA in a series of stacked planes, which generates a three-dimensional (3D) structure with a sealable entrance and a total volume in the picoliter scale. Demonstrated using first generation microcontainers, populations of *Pseudomonas aeruginosa* engaged in quorum sensing behaviors within populations of high local density (≥10^4^ cells). Furthermore, populations with as few as ∼150 cells, but at high density became gentamycin resistant, replicating a biofilm phenotype (Connell et al., 2010). This example underscores the importance of communication in communities through quorum sensing and other mechanisms. Communication mechanisms in bacterial communities have been extensively reviewed elsewhere (e.g., see Parsek and Greenberg, 2005; Waters and Bassler, 2005; Atkinson and Williams, 2009) and we refer the reader to these and other sources for in-depth discussion of intercellular communication. The MPL technology has recently expanded to a point where bacterial communities can be “3D printed” with defined shape and structure. In bacterial 3D printing cultures are suspended in warm gelatin, which is then cooled to induce gelation. Cells of interest are encapsulated in microcontainers using MPL and uncrosslinked gelatin is washed away (Connell et al., 2013). 3D printing was used to investigate interspecies interactions at low cell number. For example, it was demonstrated that encapsulated populations of *S. aureus* were protected from exogenously applied β-lactam antibiotics by a surrounding shell of *P. aeruginosa* cells that produce β-lactamases (Connell et al., 2013; **Figure 5A**). An exciting extension of 3D bacterial communities has been demonstrated by coupling microcontainers to sensitive analytical instruments including scanning electrochemical microscopes (SECM). In SECM an ultramicroelectrode is used to sensitively measure local concentrations of redox active molecules. For example, SECM has been used to measure H_2_O_2_ production in mixed species biofilms of *Aggregatibacter actinomycetemcomitans* and *Streptococcus gordonii* (Liu et al., 2011). SCEM has also been coupled with 3D printing to measure real-time production of pyocyanin by *P. aeruginosa* (Connell et al., 2014). By coupling 3D printed communities with sensitive instruments like SECMs or mass spectrometers, leveraging the power of bacterial genetics, and applying insights gained from agar plate studies, we will be able to better understand community interactions at more realistic cell densities.

While new technologies have enabled examination of bacterial communities at small spatial scales, studies of natural communities remains challenged by the difficulty in culturing many bacteria from the environment in the laboratory. It is estimated that only ∼0.1-1% of bacterial cells from environmental samples will grow under laboratory conditions (Amann et al., 1995). This “great plate count anomaly” can be partially circumvented by careful adjustments in how bacteriological media is prepared, but still large numbers of organisms are not currently cultivatable due to unknown nutritional requirements that are likely supplied through syntrophic interactions with neighbors (Morris et al., 2013; Tanaka et al., 2014). The newest advancement for culturing difficult bacteria has been the development of devices such as the iChip (Nichols et al., 2010). The iChip is a device that contains many channels sandwiched between two semi-permeable membranes. Environmental samples, such as soil, are diluted and loaded into the iChip so that channels contain a single cell. Then the device is returned to the environment at the sampling location (**Figure 5B**). Bacteria within the channels of the iChip exchange metabolites with their native environment through the semi-permeable membranes. These conditions promote formation of isolated colonies within the device’s channels, which in some cases overcome the key limitation to laboratory growth. In other words, once environmental isolates form substantial colonies they are often amiable to further manipulation in the laboratory (Ling et al., 2015), perhaps because once cells reach a critical density they are no longer reliant upon support from their neighbors to grow. Recently, the iChip was used to identify teixobactin, a new antibiotic produced by a previously uncultured bacterial species (Ling et al., 2015). Clever techniques that enable us to culture more bacteria will also assist in determining how bacterial communities interact. Despite these advances, it is probable that some bacterial species will remain a challenge for laboratory cultivation. The use of culture-independent techniques is necessary to understand how these organisms engage in competition and affect community dynamics.

## Metagenomics Reveals How Bacterial Competition Promotes Healthy Microbiota and Function

Currently, some of the most fervent areas of research in microbiology are investigations into how microbiota influences the health of plants and animals (Ley et al., 2008; Lebeis et al., 2015; Lloyd-Price et al., 2016). Tremendous interest focuses on characterizing and understanding the human microflora and its influences on health and disease. For example, inoculation with bacterial communities is essential for normal gut development. The GI tracts of Germ-free mice have defects in antibody production, lymphoid tissue development, and Peyer’s patch formation (Round and Mazmanian, 2009). Furthermore, antibiotic treatment can alter bacterial community composition and lead to diseases such as colitis caused by *Clostridium difficile* (Becattini et al., 2016). At birth, bacteria from the mother’s fecal, skin, and vaginal microbiota colonize the infant human (Mueller et al., 2015). In newborns it is thought that aerobic and facultative anaerobic bacteria first colonize the GI tract and consume the available oxygen, which converts the tract into a more anaerobic environment (Adlerberth, 2008). Concurrent with oxygen consumption, the composition of the gut bacterial community shifts from being predominantly colonized by aerobes to containing primarily facultative and obligate anaerobes. This change is reflected in *E. coli* isolated from an infant from birth to two years of age. Earlier isolates of *E. coli* have a higher growth rate under aerobic conditions but lower growth rate under anaerobic conditions when compared to later isolates (de Muinck et al., 2013).

The commensal bacteria community in the gut provides the host with an important service by preventing the colonization of pathogens (Buffie and Pamer, 2013; Britton and Young, 2014; Singh et al., 2014; Buffie et al., 2015). For pathogens to circumvent resistance to colonization, they employ a number of competitive mechanisms to antagonize the commensal microbiota. One strategy that pathogenic bacteria use to invade the gut bacterial community is to take advantage of metabolic resources that are inaccessible to other species. For example, unlike commensal bacteria, the pathogen *Vibrio cholerae* is able to use the abundant sialic acid, found on mucins, as a sole carbon source (Almagro-Moreno and Boyd, 2009). In a more extreme example *Salmonella enterica* serotype Typhimurium (*S.* Typhimurium) triggers inflammation during infection, which results in perturbations to the commensal community (Barman et al., 2008). The inflamed GI tract produces tetrathionate that *S.* Typhimurium can use as an electron acceptor during the metabolism of ethanolamine (Thiennimitr et al., 2011). Inflammation damages the host microbiota, reducing competitors. Because other species can’t use ethanolamine as a carbon source, *S.* Typhimurium gains a competitive advantage in colonizing the GI tract (Thiennimitr et al., 2011). However, the commensal bacterial community is not defenseless against assault. Like *S.* Typhimurium, commensal bacteria influence their external environment to promote their survival. The type VI secretion system (T6SS) of *V. cholerae* is activated by mucin and its activity is modulated by bile salts. Several commensal bacterial species including *Bifidobacterium bifidum* convert bile salt species into deoxycholate, which represses the *V. cholerae* T6SS (Bachmann et al., 2015). Commensal species also engage in interference competition. *Bacteroides fragilis* produces an antimicrobial protein BSAP-1 with a membrane complex/perforin domain, which is incorporated into extracellular vesicles (Chatzidaki-Livanis et al., 2014). Additionally, *B. fragilis* produces a T6SS, which has been demonstrated to function *in vivo* (Chatzidaki-Livanis et al., 2016). Commensal strains of *Enterococcus faecalis* produce a plasmid-encoded bacteriocin, which allows them to displace other colonized *E. faecalis* strains including multidrug-resistant strains (Kommineni et al., 2015). In summary, the gut bacterial community is a highly competitive environment, but most of our insights into its competitive mechanisms result from interaction studies in the laboratory. Given that the gut microbiota consists of on average between 500 and 1000 species (Human Microbiome Project Consortium, 2012) new approaches are necessary to identify competitive interactions of interest within this community.

Some of our initial insights in characterizing community diversity were through denaturing gradient gel electrophoresis (DGGE). In DGGE PCR amplified products, usually 16S rDNA, are subject to a gradient of denaturing conditions, which yields a banding pattern on a gel (Muyzer and Smalla, 1998). The DGGE banding pattern is used as a fingerprint for a bacterial community’s composition but is limited by low resolution among closely related species. The sequences of DNA bands must be determined individually (Nocker et al., 2007). High throughput sequencing (HTS) has transformed microbial ecology in recent years. One of the primary applications of HTS in microbial ecology is to characterize metagenomes, which are the DNA sequences present in an environmental sample. In particular, metagenomic approaches have been widely applied to the study of the human gut microbiota (Human Microbiome Project Consortium, 2012; Clavel et al., 2016; Moore-Connors et al., 2016).

**FIGURE 6:**
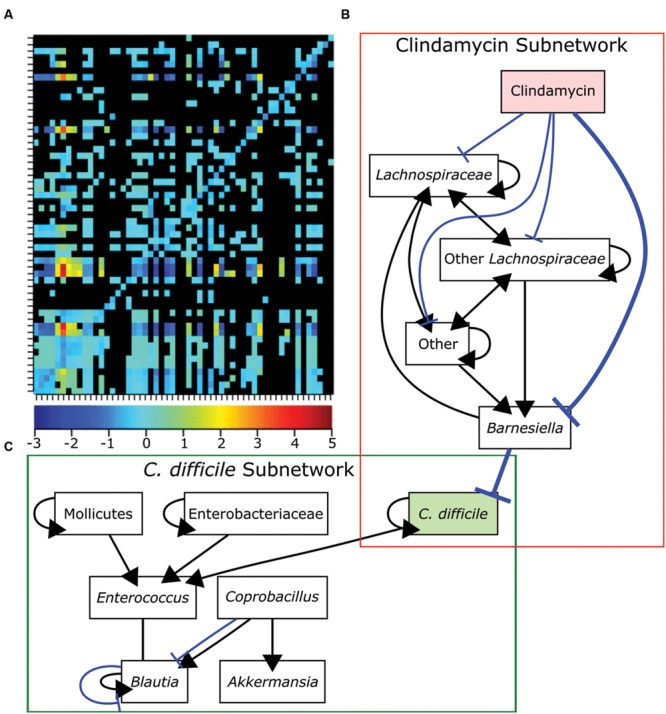
**Bacteria competition in the gut microbiome. (A)** Genus-level bacterial interactions in a human microbiome as predicted from community time series analysis. Each cell in the grid represents the effect of the x-axis bacterial genus on the corresponding y-axis bacterial genus. The heat map shows the strength and direction of each interaction with positive values representing cooperative behaviors and negative values representing competitive behaviors. The zero indicates amensal interactions and black cells indicate no significant interaction. **(B-C)** A dynamic Boolean network model of interactions in the GI tracts of mice treated with clindamycin alone **(B)** or mice exposed to *Clostridium difficile* after clindamycin treatment **(C)**. A single edge between *Barnesiella* and *C. difficile* connects the networks and is emphasized in bold. Positive interactions are shown in black with arrowheads designating the interaction direction. Negative interactions are shown in blue with T-ends designating the interaction direction. Panel **(A)** was reproduced from Trosvik and de Muinck (2015) and panels **(B,C)** were adapted from data in Steinway et al. (2015) under the terms of the Creative Commons Attribution License.

Many initial metagenomic studies focused on cataloging the bacterial diversity in healthy and diseased individuals then correlating the presence or absence of different bacterial species to each state. Metagenomic analyses have the potential to uncover the composition and connectivity of species in a bacterial community. However, by only using single snapshot binary comparisons of two community states, it is not possible to ascertain how members of the community interact. To infer interactions from metagenomic data it is necessary to following the composition of a community over a sufficiently long period of time with high temporal resolution (Trosvik et al., 2015). Only recently have these data existed and been available for generating models. Community time series analysis was recently applied to metagenomic data acquired from four humans at high temporal resolution to identify foundation and keystone taxa as well as interactions across the whole community (Trosvik and de Muinck, 2015). Briefly, linear regression was used to determine the interaction strength and direction for each pairwise genus combination over time. Consistent with previous findings for all four individuals, most of the interactions between genera were either amensal or competitive (Trosvik and de Muinck, 2015; **Figure 6A**). Community time series analysis was also recently used to determine how antibiotic treatment leads to susceptibility to *C. difficile* infection (Steinway et al., 2015). The abundances of bacterial genera were measured over 23 days in control mice, clindamycin-treated mice, and clindamycin-treated mice exposed to *C. difficile* (Stein et al., 2013). To generate a network model, Boolean rules were inferred by determining how the abundances of bacterial genera differed over time in relation to each other. This network contained two subnetworks connected by a single edge. The first subnetwork contained genera negatively influenced by clindamycin while the other contained other species including *C. difficile* (**Figures 6B,C**). The single edge that connected *Barnesiella* in the first subnetwork to *C. difficile* suggested that the latter is competitively inhibited by the former. Clindamycin treatment relieves this repression. Subsequent experiments demonstrated that *C. difficile* growth rate was reduced in the presence of *Barnesiella intestinihominis* or its spent media, suggesting that competition between these two organisms may be important for the host’s health (Steinway et al., 2015).

Metagenomics has become a powerful approach in determining the composition and connectivity of bacteria in a community. However, there are limits to these approaches for understanding community dynamics. The first is that traditional HTS library preparation protocols do not distinguish between DNA isolated from live or dead cells. This limitation could be circumvented through the application of photoactive DNA intercalators such as propidium monoazide or ethidium monoazide bromide, which crosslink to DNA and render it insoluble during standard extraction. Importantly these DNA intercalators are excluded from cells with active membranes (Nocker et al., 2006; Delgado-Viscogliosi et al., 2009). Unfortunately, the use of DNA intercalators may be limited in studies investigating bacterial communities from the GI tract because *in situ* application of the intercalators is not feasible, and the viability of obligate anaerobes may be underestimated if the intercalators are applied after samples collection in an aerobic environment. Alternatively, RNA can be isolated from a bacterial community and sequenced. This approach, known as metatranscriptomics, may better reflect a community’s composition because metabolically active cells constantly transcribe RNAs including ribosomal RNAs (Gosalbes et al., 2011). One can also use metatranscriptomics to analyze mRNA content, and subsequently the community’s transcriptional activity. This approach has some technical difficulties including the need to enrich for mRNA over rRNA without introducing sequence biases (Bikel et al., 2015). Identifying organisms relies on the 16S sequence, which also has a limited resolution. While the 16S sequence can be used to identify an organism to the level of genus or species it is not informative for the remainder of the gene content in the genome. For example, a given *E. coli* isolate only has ∼20% DNA sequence in common with all other *E. coli* (Lukjancenko et al., 2010). Some pathogens like *V. cholerae* require a prophage for their virulence (Davis and Waldor, 2003). Bacteriocin production is not common among all strains of a species and each strain’s genome may encode a unique suite of bacteriocins, which may be encoded on prophages (Bogaardt et al., 2015; Moon et al., 2015; Miller et al., 2016). Thus, the 16S sequence is a poor proxy for understanding the competitive and metabolic potential of a strain. Metabolomic and proteomic approaches have been integrated with metagenomics to circumvent the limitations of 16S sequence alone (Daniel et al., 2014; Tong et al., 2014; Zhang et al., 2015). Lastly, all sequencing based approaches are limited by sampling. In most gut microbiome studies the nucleic acid is extracted from a fecal sample. As the bacteria in a fecal sample are being removed from a body their composition may not be a true reflection of the activities occurring in the proximal GI tract. Likewise, in sequencing nucleic acid from a large environmental sample, e.g., grams of soil or milliliters of lake water, many of the bacterial species may have never encountered each other naturally. We have highlighted some technological limitations of metagenomics applied to community dynamics in order to focus attention toward areas for innovation, because metagenomic approaches are revolutionary for determining the composition and connectivity of a bacterial community. By wedding models generated from metagenomic data to complementary cultured-dependent experiments we will gain deeper insights into the specific interactions that undergird community.

## Modeling Reveals that Competition and Spatial Structure Reinforce Biodiversity in Bacterial Communities

Biodiversity is a parameter studied in both macro- and microbiological communities. Biodiversity is simply defined as the variety of life in a particular ecosystem and it is often expressed as the number and abundance of different genes or species that are present (Swingland, 2001). The health of an ecological community is often described as a function of both its stability and biodiversity. Presumably, a community with higher levels of biodiversity has more of its total potential niches occupied, which results in a greater sum usage of resources than a community with less biodiversity (Hunting et al., 2015). Additionally, communities with more niches occupied are better positioned to resist external invaders (Mallon et al., 2015).

Beyond a measure for the potential fitness of community member’s, changes in bacterial biodiversity have consequences in diverse processes ranging from human health to bioremediation. Loss of bacterial biodiversity can result in the dysfunction and eventual collapse of a community, e.g., *C. difficile* infection. Often the only effective treatments for recurrent *C. difficile* infection are either surgical removal of the diseased tissue and/or fecal transplant to replenish the GI tract with a functional microbial community (Petrof et al., 2013; Ofosu, 2016; Pamer et al., 2016). While we have emphasized GI tracts as an example biodiversity is also important and relevant in broader cases such as bioremediation (Dell’Anno et al., 2012; Venail and Vives, 2013a,b).

How biodiversity is established and maintained in communities is not well understood and is a major question in ecology. Bacterial communities provide an excellent experimental model to address this and other fundamental questions. In some part biodiversity may be maintained due to apparent cooperation amongst community members resulting from concurrent metabolic specialization and adaptive gene loss (see above). Competition is prevalent in bacterial communities (Pérez-Gutiérrez et al., 2013) and is the major mode of interaction among bacteria (Foster and Bell, 2012). Thus, to understand bacterial communities we must ask how competition affects a community’s biodiversity and function. Intuitively, competition may have negative consequences on biodiversity. It has been reported that increased antagonism results in collapsed communities (Becker et al., 2012). Interactions in two species systems containing an antagonist (e.g., an antibiotic producer) and a susceptible target (e.g., an antibiotic sensitive strain) are inherently unstable: one species will drive the other to extinction, depending on relative metabolic costs (Blanchard et al., 2014). However, in the natural environment bacterial communities rarely, if at all, consist primarily of only two species.

Investigation into the causes of biodiversity provides a case example of the complementarity of the experimental models and modeling approaches to studying bacterial communities. Initial mathematical models posed the question: if a two species community is unstable, what about a three species community? By invoking the presence of a third species, rock-paper-scissors (RPS) dynamics emerge (Czárán et al., 2002; Kerr et al., 2002; Kelsic et al., 2015). In the two species community, each species has the state of antibiotic-sensitive (S) or antibiotic-producer (P). If, for example, members of the community can acquire a “resistance” state (R), which protects them from the antibiotic but is more metabolically costly than the “sensitive” state (P > S, S > R, and R > P) then non-transitive RPS dynamics with cyclic dominance can occur given the proper initial conditions (Czárán et al., 2002). For example, the colicins are proteinaceous antibiotics produced by *E. coli* and have narrow-spectrum activity targeted against closely related organisms (Braun et al., 1994; Riley and Gordon, 1999). Colicins are of particular interest in bacterial ecology because *E. coli* strains with differences in colicin production compete with each other under RPS dynamics (Kerr et al., 2002). Using two-dimensional cell automata models, the three species community was predicted to be stable and subsequently stability was experimentally demonstrated (Kerr et al., 2002).

**FIGURE 7:**
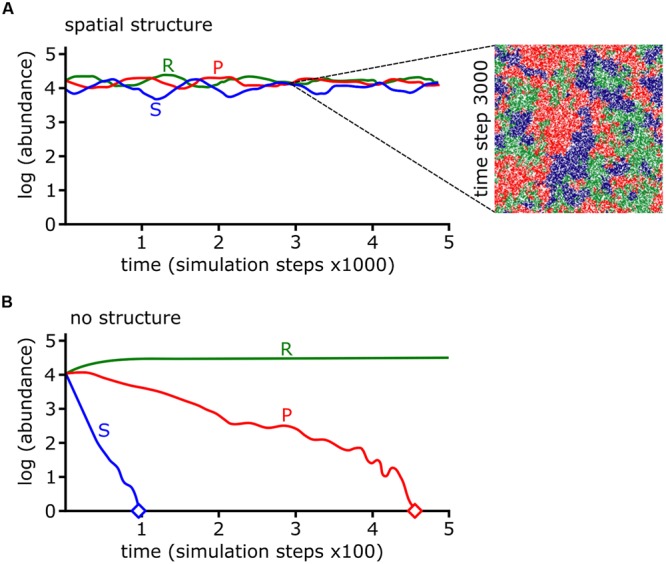
**Rock-paper-scissors (RPS) dynamics and spatial structure stabilize bacterial communities. (A,B)** The log abundances of colicin producing (P), colicin sensitive (S), and colicin resistant (R) strains from cellular automata simulations are shown over simulation time. The abundances of each strain are stable if interactions occur locally (i.e., with spatial structure) **(A)** but under well-mixed conditions (i.e., with no spatial structure) the P and S strains go extinct, indicated by diamond symbol **(B)**. The inset in **(A)** shows a snapshot of the simulation at time step 3000. Data and images were adapted by permission from Macmillan Publishers Ltd (Kerr et al., 2002), Copyright © 2002, Rights Managed by Nature Publishing Group.

The colicin RPS model predicted that a sensitive species can survive in the presence of a producer as long as the community is spatially organized (Kerr et al., 2002; **Figure 7**). In other words, competition promotes biodiversity but spatial structure is required to limit opportunities for antibiotic sensitive strains to encounter antibiotic producers. Spatial structure may result from the physical environment. For example, the uneven distribution of microcolonies on leaf surface (Monier and Lindow, 2005), low connectivity of pores in soil (Carson et al., 2010), and patchy landscapes (Hol et al., 2016) can limit the opportunities for antagonists to kill their targets. Biotic factors such as the heterogeneous distribution of bacteria in the natural environment also facilitate the formation of spatial structure. For example, 78 thermoresistant strains, mostly *Bacillus* spp., were isolated from across five positions in the same lake with very different species distributions between each site (Pérez-Gutiérrez et al., 2013). Pairwise interactions for each of the 78 strains revealed a high degree of antagonism, though more severe antagonism typically occurred between strains isolated from different sites (Pérez-Gutiérrez et al., 2013). A cell automata model was built using the observations from all possible pairwise interactions between the 78 strains. The model predicted high biodiversity with different strains occupying spatially segregated patches across the surface (Zapién-Campos et al., 2015). The model also predicted that survival of weaker bacteria is dependent upon being shielded from aggressors by patches of a third bacterial strain (Zapién-Campos et al., 2015). This prediction is borne out in a model three species biofilm formed by isolates of soil bacteria. Pyocyanin-sensitive *Brevibacillus borstelensis* is protected from the pyocyanin producer *P. aeruginosa*, if both species are separated by pyocyanin resistant *Raoultella ornithinolytica* (Narisawa et al., 2008). Competitive interactions between bacterial species may form the underlying foundation of spatial structure, which is necessary to maintain biodiversity for bacterial species competing under RPS dynamics.

In many cell automata models RPS-mediated spatial structure emerges as moving spiral patterns between the three species (Reichenbach et al., 2007; Kelsic et al., 2015). If model parameters are adjusted and cell mobility increases above a threshold value then the wavelength of spirals exceed the grid space of the cell automata model, increasing the probability that species go extinct and reduce biodiversity (Reichenbach et al., 2007). Experimentally, increased mobility can be modeled by growth in liquid batch culture or by random distribution replica plating of mixed species lawns on agar plates. In either case, increased mobility drives both the sensitive and producer strains extinct while the resistant strain persists (Kerr et al., 2002). The models previously described considered that interactions between cells were local, i.e., occurring between adjacent cells on the grid. In interactions resulting from diffusion, the effect of cell mobility is dependent upon the distance between two cells relative to diffusion of the effector (Song et al., 2009). Below a first critical point where the cells are in close enough proximity there is no effect of mobility on biodiversity because the physical distance is less than the limit of diffusion. Likewise, at distances above a second critical point there is no effect on biodiversity, because cells are too far away to interact (Song et al., 2009).

We have presented several lines of evidence, derived both from theoretical modeling and experiments, demonstrating that spatial structure promotes biodiversity while mobility hinders it. Mathematical models are incredibly powerful tools to investigate biological phenomena, but they are limited by our current understanding of individual systems. Models attempt to capture many biological variables and place them into well-defined rules that govern how systems behave. For instance, many RPS-based models consider the antibiotic as a singular entity, i.e., the antibiotic producing strain produces a single antibiotic. However, we know, for instance that soil bacteria including *Bacillus, Myxococcus*, and *Streptomyces* that each species produces numerous specialized metabolites with antibiotic activity or competitive functions that extend beyond antibiosis (Chater, 2006; Weissman and Müller, 2010; Sansinenea and Ortiz, 2011; Stubbendieck and Straight, 2016). Though some models allow RPS dynamics to occur with respect to a larger number of antibiotics (e.g., see Czárán et al., 2002), the outcomes of interactions are mostly focused on survival or death. Other behaviors, including retaliation, may be common features in bacterial communities that are not captured by models. Strains of *P. aeruginosa* that suffer attack by T6SS from *Vibrio cholerae* reciprocate by striking, and often killing, the offending cell with their own T6SS (Basler et al., 2013). *Escherichia coli* cells that are exposed to a competitor’s colicins induce expression of their own colicins genes. This colicin expression is proportional to the inducing colicin’s efficacy (Majeed et al., 2013). In mixed species interactions *E. coli* producing weaker colicins survive better than strains producing stronger colicins, because the weaker colicins do not trigger a response in their competitors (Majeed et al., 2013). While many subtler details may be lost in simulation experiments, modeling has predictive power in understanding the dynamics of bacterial communities, which have been demonstrated experimentally. Naturally, incorporation of more mechanistic studies will improve our models and vice versa, enabling a deeper understanding of bacterial communities.

## Conclusion

**FIGURE 8:**
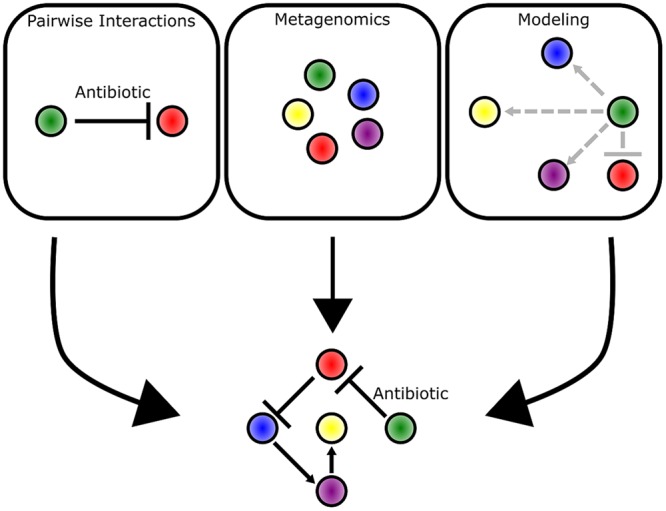
**Multiple approaches complement each other and provide different insights into bacterial communities.** Pairwise interactions provide mechanistic detail into the competitive mechanisms employed by bacteria. Metagenomic approaches enable identification of bacteria species and their abundance in a community as represented by different colored circles. Mathematical modeling approaches predict the strength (indicated by arrow length) and direction of interactions that occur between bacterial species. The three approaches complement each other and provide the clearest picture into the underlying dynamics of a bacterial community.

Bacteria live in communities that range from aggregates of a few cells to collections of billions of cells and every scale in between (**Figure 1**). By breaking down and investigating communities over a wide range of scales, we are better able to understand fundamental principles of bacterial ecology. This includes deciphering the mechanisms of pairwise interactions, as well as identifying the species composition of a bacterial community. Through the integration of complementary experimental and theoretical approaches, the underlying foundation of dynamics in larger scale communities is revealed (**Figure 8**). Moving forward, the application of both approaches will be instrumental in garnering new insights into how bacterial communities influence all facets of lives on Earth.

## Author Contributions

All authors listed, have made substantial, direct and intellectual contribution to the work, and approved it for publication.

## Conflict of Interest Statement

The authors declare that the research was conducted in the absence of any commercial or financial relationships that could be construed as a potential conflict of interest.
